# Geochemistry and Sr, S, and O stable isotopes of Miocene Abu Dhabi evaporites, United Arab Emirates

**DOI:** 10.1016/j.heliyon.2023.e16033

**Published:** 2023-05-05

**Authors:** Ahmed Gad, Osman Abdelghany, Hasan Arman, Bahaa Mahmoud, Ala Aldahan, Safwan Paramban, Mahmoud Abu Saima

**Affiliations:** a United Arab Emirates University, College of Science, Geosciences Department, P. O. Box: 15551, Al Ain, United Arab Emirates; b Ain Shams University, Faculty of Science, Geology Department, 11566, Cairo, Egypt

**Keywords:** Evaporites, Gypsum, Geochemistry, ^87^Sr/^86^Sr, Isotopes, Miocene, Abu Dhabi

## Abstract

This study investigates for the first time the subsurface Miocene evaporite facies (Gachsaran Formation) in Abu Dhabi, United Arab Emirates. Forty-five evaporite rock samples were selected for petrographic, mineralogical, and geochemical investigations and stable isotope analyses to decipher their origin and constrain their age. Secondary gypsum with anhydrite relics dominates the investigated evaporitic rocks, with minor amounts of clays, dolomicrite, Fe/Ti oxides, and celestite. These samples are characterized by their excellent purity and low variability in geochemical composition. The distribution of trace element concentrations is significantly influenced by continental detrital intake. The main focus of the study is to determine the strontium, sulfur, and oxygen stable isotope compositions. The measured ^87^Sr/^86^Sr values of 0.708411–0.708739 are consistent with Miocene marine sulfates and indicate ∼21.12–15.91 Ma (Late Aquitanian-Burdigalian). The δ^34^S and δ^18^O values are 17.10‰–21.59‰ and 11.89‰–19.16‰, respectively. These values are comparable to those of Tertiary marine evaporites. The relatively low values of δ^34^S suggest that non-marine water possesses little influence on S distribution. The geochemical composition and Sr, S, and O isotope distributions of the Abu Dhabi gypsum facies from the Gachsaran Formation reveals that their source brines were marine (coastal saline/sabkha) with subordinate continental input.

## 1 Introduction

Evaporites reflect the ancient climatic, geographical, environmental, and geochemical changes occurring in sedimentary basins [1]. Facies analyses combined with mineralogic, and petrographic examination of evaporitic units can disclose depositional environments and paleoclimatic conditions at the time of formation [2,3]. Because evaporite rocks are extremely susceptible to post-depositional alterations, depositional and diagenetic environments may affect the elemental composition of their minerals [4]. A large amount of information regarding paleosalinity, initial brine composition, depositional basin water depth, paleotemperature, evaporite origins, and diagenetic processes have been based on the geochemistry of major and trace elements [[5], [6], [7]]. Moreover, a significant contribution for determining the origin, marine vs non-marine, of ancient evaporites and the influence of different water types on the depositional basin may be acquired from the use of strontium (^87^Sr/^86^Sr), oxygen (δ^18^O_sulfate_), and sulfur (δ^34^S_sulfate_) isotope data [[8], [9], [10], [11]]. Karakaya et al. [9] employed ^34^S and ^18^O values to deduce the contribution of the recycling-dissolution of the predeposited marine sulfates (Oligocene-Eocene), arid conditions and bacterial sulfate reduction processes on the compositions of the Miocene evaporites in the Tuz Gölü basin, Turkey. The lithologies and Sr isotope compositions of Middle Jurassic evaporites in the Qamdo and Qiangtang Basins of Eastern Tibet were compared by Fei et al. [10]. Their findings suggested that the Qiangtang Basin was mostly recharged by Jurassic seawater, whereas the Qamdo Basin was primarily recharged by continental water, with some Qiangtang Basin-derived overflow.

Understanding sedimentary evolution requires a precise dating of sediments. Age dating of evaporite deposits typically relies on palynological data and their stratigraphic relationships with other formations, which can be poorly defined [12]. Stable isotope dating (^87^Sr/^86^Sr, δ^18^O_sulfate_, and δ^34^S_sulfate_) in evaporites is probably the more accurate method for precisely dating different successions. This necessitates a thorough examination of the samples, including mineralogy, petrography, scanning electron microscopy (SEM), and stable isotope geochemistry [13].

Over the last three decades, Sr isotope stratigraphy has undergone significant advancements and has proven to be an efficient high-resolution method for sedimentary rock dating and stratigraphic correlation [[13], [14], [15], [16]]. It can be used to objectively determine the age of the sulfate deposition (gypsum and anhydrite). Such minerals naturally incorporate substantial amounts of Sr into their lattices, replacing Ca [12,17,18]. Because Sr is extremely susceptible to alteration effects (fluid-rock interactions with fluids of meteoric, diagenetic, tectonic, and metamorphic origins), caution is necessary when testing the ^87^Sr/^86^Sr ratio. The target mineral must be isolated when working with the ^87^Sr/^86^Sr ratio to prevent possible contamination from co-deposited minerals and organic materials [19]. Sulfur (δ^34^S_sulfate)_ has been used to properly assign ages to gypsum and anhydrite deposits and post-depositional alteration [4,[20], [21], [22]]. Oxygen stable isotope composition (^16^O and ^18^O) provides information on digenetic alterations in closed or opened system with more significant variations in δ^18^O in open system [23]. Shen et al. [23] attributed the complex O isotope compositions of sulfates minerals in the Lanping-Simao Basin (China) to sulfate reduction, re-oxidation, and reservoir impact processes. Surakotra et al. [4] ascribed the relatively negative value of ^18^O in sulfate deposits of the Loei-Wang Saphung (Thailand) to meteoric alteration during subaerial exposure of the sections.

Most of the researches on Abu Dhabi evaporites have been focused on the recent sabkha deposits regarding sedimentological [24], mineralogical [25], geochemical properties [26], and environmental and engineering characteristics [[27], [28], [29]]. The petrography and dating of ancient evaporite units have received little attention, and their sedimentological–petrographic, mineralogical, and geochemical characteristics have not been thoroughly investigated. These rocks were dated based on their stratigraphic position (stratigraphic age) and assigned to the Gachsaran Formation (Fars Group) referred to the Lower Miocene [29]. The Gachsaran Formation is widespread in Saudi Arabia, Kuwait, the United Arab Emirates (UAE), and Qatar. It widely outcrops from the Zagros Belt zone in southern Iran to northern Iraq and northeastern Syria [30]. Biostratigraphic studies using different biozones have indicated high variability in the age of the formation spanning the Burdigalian to the Tortonian [[31], [32], [33]]. However, the precise age of the Miocene Abu Dhabi evaporitic unit remains unknown; thus, aspects of their sedimentation history are not sufficiently detailed. Therefore, the objectives of this study are to provide a comprehensive analysis of the petrographic and geochemical characteristics of the subsurface evaporite facies in Abu Dhabi and to define their deposition evolutive history and detail the Lower Miocene age.

## 2 Geological setting

**Fig. 1:**
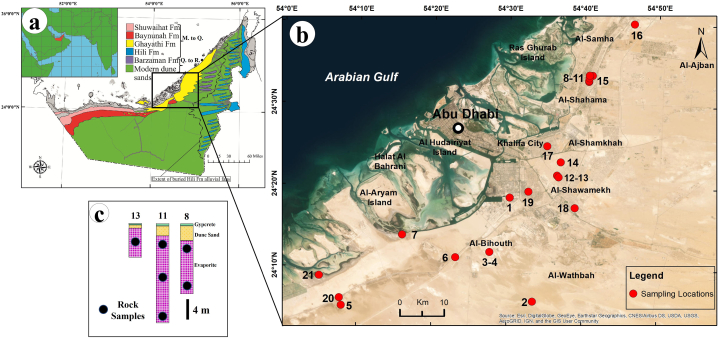
a) Simplified geological map of UAE [35] (M = Miocene; Q = Quaternary; R = Recent); b) enhanced google earth image of Abu Dhabi showing subsurface sampling locations; and c) representative stratigraphic logs for sampling locations 8, 11, and 13.

Surface geological mapping (Fig. 1a) shows four major successions that range in age from the Miocene to Quaternary (Neogene): the Shuweihat and Baynunah Formations (fluvial sandstones), Ghayathi Formation (palaeodune sandstones), Barzaman and Hili Formations (fluvial fanglomerates and sandstones), and Quaternary to recent aeolian dune sediments [34,35]. Quaternary deposits dominate on land of the Abu Dhabi region. These deposits are underlain by a thick succession of the Lower Miocene Gachsaran Formation (Fars Group) [29,36]. This Formation is composed of interbedded mudrocks, limestones, and evaporitic rocks. It conformably overlies the Upper Oligocene–Lower Miocene Asmari Formation (dolomitic and nummulitic limestone, calcareous mudstone, and anhydrite) [36]. The Abu Dhabi onshore Gachsaran Formation (Lower Miocene) was subdivided into three members. The lower member formed a salt wedge recorded only in the depocenter and pinched out on the flank of the fore bulge. The middle member is dominated by a continuous lateral series of organic-rich shale, argillaceous limestone, and subordinate anhydrite. The upper member is composed of a stack of alternating bed sets of evaporite and argillaceous limestone [37]. Evaporite facies are encountered throughout Abu Dhabi but are most observable on the eastern side of Jabal Hafit and in several subsurface sections and quarries.

## 3 Sampling and analyses

Detailed field investigations of subsurface evaporite rocks were conducted at twenty-one different locations in Abu Dhabi (Fig. 1b). Evaporite rock samples were collected from temporary excavations and boreholes that reached depths up to 20 m. In this study, forty-five evaporite rock samples were collected. The evaporite beds are mostly massive, and alabastrine is interbedded with thin beds of marl and mudstone. In some cases, it appears as a thinly laminated to nodular whitish to creamy gypsum-interbedded grayish clay-rich mudstone.

The evaporite samples were finely ground into powder (<63 μm), and the bulk samples' mineralogical composition was identified by X-ray powder diffraction (XRD) (X'Pert Pro PANaLytical X-Ray with Monochromator, Cu-radiation (λ = 1.542 Å) at 45 kV, 35 mA, and scanning speed 0.02°/s). Standard thin sections were examined using a polarizing microscope equipped with a digital camera (Nikon, LV 100 N POL with NIS Elements imaging software).

X-ray fluorescence (XRF) elemental analysis was conducted for powder (<74 μm) samples (PW 2404 with five analyzing crystals). With an accuracy of 99.99% and a confidence limit of 96.7%, the software programs Super Q and Semi Q were used to calculate the concentrations of the tested major elements (oxides%) and trace elements (ppm).

Individual microscopically picked mineral crystals were analyzed using scanning electron microscopy (SEM) and energy dispersive spectrometry (EDS) (SEM/EDX, XL 30 ESEM, Philips Co., low vacuum condition, accelerating voltage 30 kV, working distance Z10, 60-s counting time, and imaging with a BSE detector).

The oxygen (^18^O), sulfur (^34^S), and strontium (^87^Sr/^86^Sr) isotopic compositions were measured on hand and microscopic-picked gypsum crystals at Stable Isotope Geochemistry Laboratory (SIGL) within Earth and Environmental Sciences at the University of Queensland, Australia. The ^18^O in BaSO_4_ was analyzed using a Thermo TCEA and reported with respect to the Vienna Standard Mean Ocean Water (VSMOW). All samples were precipitated as BaSO_4_ and then oven-dried. Samples were weighed into silver capsules, placed in a vacuum oven at 80° overnight, and loaded into the zeroblank autosampler; next, any ^18^O contamination from moisture and atmosphere was removed. NBS-127, SMOW, and SLAP were used as international standards, with 3-point normalization to the VSMOW-SLAP scale. The long-term standard deviation for carbonate oxygen isotope is ±0.14‰. The δ ^34^S was determined using an elemental analyzer (EA-IRMS, Elementar, Vario Isotope Cube coupled to a PrecisION isotope ratio mass spectrometer) and reported with respect to the standard, Vienna Canyon Diablo Troilite (VCDT). Calibration was conducted using 3-point normalization with international silver sulfide standards IAEA-S-1 (−0.30‰), IAEA-S-3 (−32.3‰ ±0.2), and sulfate NBS-127 (21.12‰) and an unknown S-MIF laboratory pyrite standard. The analytical error is ±0.3‰.

Samples dissolved in 2 N HNO_3_ were loaded onto columns pre-filled with 0.19 ml Eichrom Sr-spec resin (50–100 m) for ^87^Sr/^86^Sr ratio measurement. Sr was extracted from the columns and collected with 2.8 ml 0.05 N HNO_3_. Sr concentrations were measured using a Thermo X-series II quadrupole Inductively Coupled Plasma Mass Spectrometer (ICP-MS). Next, for each sample, a 3 ml dilute aliquot in 2% HNO_3_ (vol/vol) was prepared for the analysis of Sr isotopic compositions using a Nu Plasma HR Multi-collector ICP-MS. Mass fractionation was corrected by normalizing to ^86^Sr/^88^Sr = 0.1194. Calibration was performed using an SRM-987 standard solution, which was measured for every five samples with a long-term analytical precision of (±0.000006 2σ).

The measured ^87^Sr/^86^Sr values were used to calculate the numerical age according to the following equation [38,39]:

$$Numerical\;age\;\left(Ma\right)=\frac{87Sr{/}^{86}Sr-0.70974}{-0.0000629}$$

## 4 Results and discussion

### 4.1 Mineralogy and petrography

**Fig. 2:**
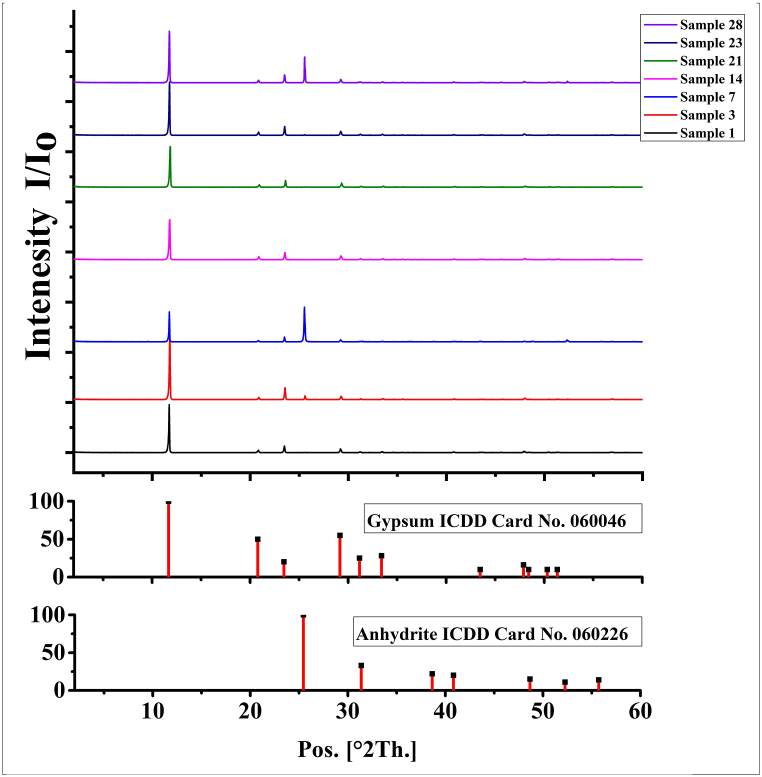
XRD diffractograms of representative evaporite rock samples.

The mineralogical composition of Abu Dhabi subsurface evaporite rock is relatively homogeneous. The XRD diffractograms of the studied evaporite samples (Fig. 2) clearly reveal that the predominant sulfate phase is gypsum, with minor amounts of anhydrite. Notably, no phyllosilicates, oxide minerals, or celestites are detected in the XRD diffractograms.

**Fig. 3:**
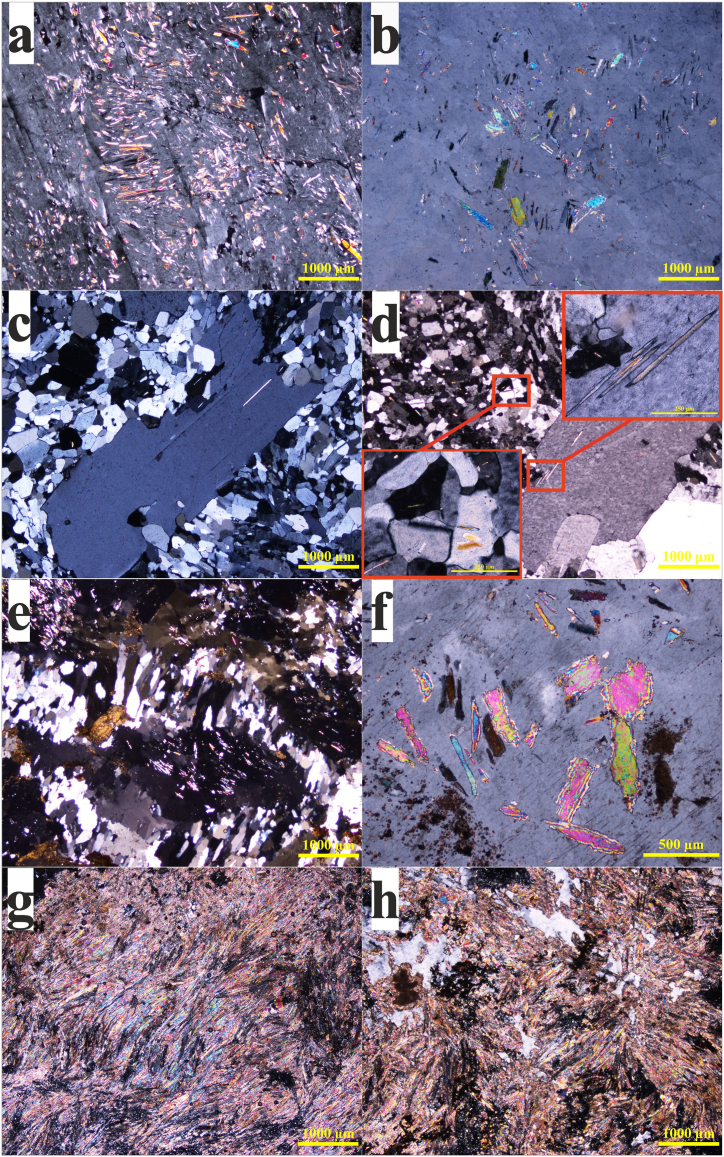
Photomicrographs (crossed polars) of subsurface evaporite rock from Abu Dhabi: a, b) poikilotopic texture of anhydrite laths in a large, bladed gypsum crystal, c, d) porphyrotopic textures; both the groundmass and the phenocryst have anhydrite lathes, e) fibrous (satin-spar) gypsum, f) irregular anhydrite crystals, g, h) anhydrite replaced gypsum crystals.

The texture of gypsum varies substantially from xenotopic (equidimensional gypsum crystals) to idiotopic, but poikilotopic and porphyrotopic textures are typically clear, preserving anhydrite relics either isolated or parallel to subparallel groups (Fig. 3a,b,c). The hydration veins of the fibrous (satin-spar) gypsum are pronounced and pervasive (Fig. 3d and e). No primary texture (selenitic-twinned gypsum crystals) was observed. Anhydrite occurred as inclusions within large gypsum crystals and irregularly etched crystals, representing relics of preexisting anhydrite (Fig. 3f and g). Only in two thin sections, anhydrite crystals were observed as a major component (Fig. 3h). The studied gypsum samples show petrographic features similar to Miocene evaporite in Ulaş-Sivas Basin, Turkey [40], and the Miocene Vilob Gypsum, Spain [20].

**Fig. 4:**
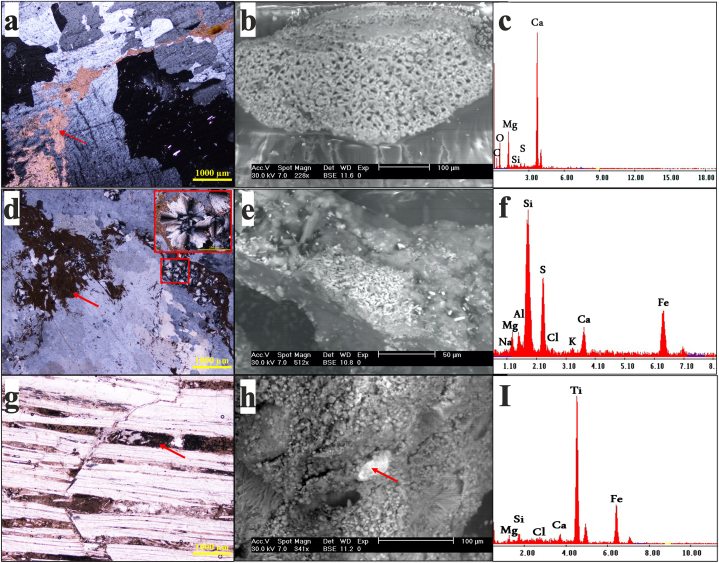
Photomicrographs (crossed polars) and SEM/EDX of subsurface evaporite rock from Abu Dhabi: a-c) dolomicrite, d-f) clay patches with spherulite silica grains, g-i) interlaminations of clays and Fe/Ti oxides.

**Fig. 5:**
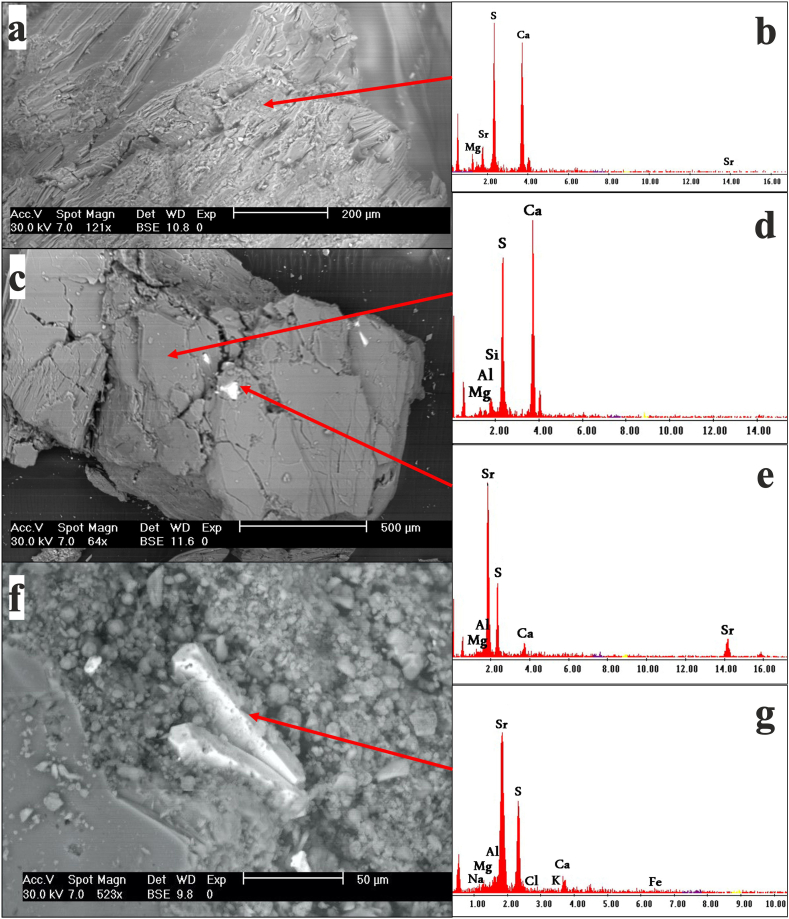
Photomicrographs of SEM-EDX analysis performed on selected samples. a, b) gypsum crystal incorporated Sr, c-e) irregular celestite crystal inclusions in gypsum crystal, f, g) prismatic celestite crystal.

The carbonate associated with this secondary gypsum was primarily dolomicrite (microcrystalline dolomite), which formed irregularly distributed masses within the rock (Fig. 4a,b,c). Some samples contain abundant clay patches with scattered silt-sized quartz grains of terrigenous origin (Fig. 4d,e,f). Occasionally, gypsum crystals show interlaminations of clays and iron and titanium oxides (Fig. 4g,h,i). Owing to its very fine-grained nature and scarcity, celestite was identified using SEM/EDX (Fig. 5). Owing to its low content and solubility, SrSO_4_ has no effect on Sr isotope determination.

Petrographic and mineralogical examinations demonstrate that the studied evaporite rocks are primarily composed of secondary gypsum (diagenetic gypsum) derived from anhydrite hydration. Primary gypsum precipitates directly from brines, retains its original texture, and is devoid of anhydrite relics [2,41]. Only high-salinity brines produce primary anhydrites [42]. This does not apply to our investigation; indirect evidence derives from the absence of halite beds in the study area or halide minerals in the investigated samples. Gypsum is dehydrated when exposed to hot, arid conditions at the surface, reacts with brine at or near the surface, is involved in burial diagenesis at deep and/or shallow depths, or is subjected to tectonic pressure [43]. Primary gypsum is converted to anhydrite through burial at varying depths depending on basin conditions [44,45]. The Miocene Abu Dhabi evaporite facies were deposited originally as gypsum in coastal salina/sabkha (laminated and nodular lithofacies). Gypsum dehydration most likely occurred during shallow-to-deep burial (early diagenesis) [44], completely transforming the primary gypsum texture into nodular anhydrite [46]. During the uplift to near-surface depths, the anhydrite changed to gypsum.

### 4.2 Geochemistry

The chemical compositions of the investigated samples were in the order of SO_3_> CaO > SiO_2_> MgO > Fe_2_O_3_ (Table 1). CaO and SO_3_ contents vary from 32.40% to 44.19% and 24.85%–46.05%, respectively. The gap between the mean and standard deviation of CaO and SO_3_, as well as the limited value of their coefficient of variation, indicates a uniform distribution of these ions, which may be related to the similar physicochemical conditions in the deposition basin. Most of the examined samples include the three main gypsum constituents CaO, SO_3_, and H_2_O in a high percentage (98%–99%), leading us to posit that the analyzed gypsum is of excellent purity. Conversely, the co-precipitated elements explain the great variability; Fe_2_O_3_ and TiO_2_ are present in nonsignificant proportions, indicating Fe/Ti oxide impurities. Because Fe and Ti are hydrolysate elements, they accumulate in fine-grained sediments [47]. In addition, the SiO_2_ and Al_2_O_3_ contents were relatively low, indicating a small influx of terrigenous (detrital) materials into the basin of deposition. Bahadori et al. [5] concluded that as the paleoenvironment of the Gachsaran Formation became shallower as a result of evaporation, the combined conditions became more oxidizing and alkaline. Fe oxides were formed and co-precipitated with Al_2_O_3_, TiO, and MgO, which scavenged the trace metals.

**Table 1:** Descriptive statistics of major oxides (%) and trace elements (ppm) in Abu Dhabi evaporite rocks (N = 25).

Major (%)	SiO_2_	TiO_2_	Al_2_O_3_	Fe_2_O_3_	MnO	MgO	CaO	Na_2_O	K_2_O	Cl	SO_3_	LOI
Min.	UDL	UDL	UDL	UDL	UDL	UDL	32.40	UDL	UDL	UDL	24.85	14.13
Max.	4.38	0.09	0.24	2.09	0.06	3.58	44.19	0.01	0.10	3.39	46.05	22.47
Mean	0.42	0.02	0.13	0.19	0.06	0.50	35.02	0.01	0.10	1.71	42.33	21.11
StD	0.86	0.02	0.09	0.44	–	0.87	2.58	–	–	2.38	3.88	1.52
CV	2.02	1.33	0.71	2.35	–	1.75	0.07	–	–	1.39	0.09	0.07
**Trace (ppm)**	**Ni**	**Cu**	**Zn**	**Rb**	**Sr**	**Zr**	**Ba**	**La**	**Co**	**Pb**	**Sn**	
Min.	UDL	UDL	UDL	2.40	247.60	38.10	35.90	7.80	26.90	UDL	3.00	
Max.	143.90	13.90	17.50	17.70	3476.90	848.00	150.60	13.30	206.90	9.90	4.80	
Mean	38.80	4.35	6.30	6.37	963.59	131.20	67.38	9.35	43.48	3.31	3.56	
StD	70.08	6.39	9.70	2.75	906.72	170.35	31.01	1.24	35.57	2.91	0.33	
CV	1.81	1.47	1.54	0.43	0.94	1.30	0.46	0.13	0.82	0.88	0.09	
PCC(CaO)	–	–	–	0.71	−0.01	0.03	0.32	0.14	0.05	–	0.54	
PCC(SiO_2_)	–	–	–	0.82	0.04	0.09	0.48	0.29	0.05	–	0.78	
PCC(Fe_2_O_3_)	–	–	–	0.81	0.06	0.12	0.49	0.28	0.02	–	0.76	
PCC(MgO)	–	–	–	0.71	0.18	0.19	0.55	0.37	−0.02	–	0.72	

UDL= Under Detection Limit, StD = Standard Deviation, CV=Coefficient of Variance, PCC=Pearson Correlation Coefficient.

Brine concentration fulfills a significant role versus Na^+^, K^+^, Mg^2+^, and Mn^2+^ co-precipitation or incorporation in gypsum under various depositional environments [6,17,47]. The exceptionally low concentration of co-precipitated Na and K in the investigated evaporite deposits suggests that the brine fluids that precipitate this gypsum deposit under equilibrium conditions have comparatively low salinities. Gypsum precipitation in equilibrium with low-salinity waters (interbedded with carbonates) would have lower concentrations of co-precipitated Na, K, and Mg than those precipitating at higher salinities (interbedded with halite) [48,49].

The analysis of the trace element concentrations revealed that the highest impurities in gypsum were Sr, Zr, Co, Ba, La, Rb, Sn, and Pb. Concentrations of V, Cr, Ni, Cu, Y, Nb, and Th were generally below the detection limit, except for a few samples with exceptionally low concentrations. Table 1 shows distinct variations in Sr concentration. Sr observed values vary from 247.6 to 3476.9 ppm. In evaporites formed in marine environments, Sr values vary between 1000 and 5000 ppm [9]. These variations in Sr concentration provide evidence for unstable salinity conditions during and/or after the formation of the gypsum deposit. Diagenetic processes led to the liberation of substantial amounts of strontium ions, and they developed local intense concentrations within these evaporitic rocks. Sr is typically present in higher concentrations in anhydrite than in gypsum [48]. Strontium is released into the solution during the hydration of anhydrite to gypsum owing to the incapacity of the monoclinic structure of gypsum to accommodate all strontium released from anhydrite [48,50]. Accordingly, some of the celestite found in the Abu Dhabi gypsum was probably formed during this diagenetic process as a by-product of the anhydrite hydration, and the elevated Sr concentration detected in some samples was related to the presence of this mineral.

**Fig. 6:**
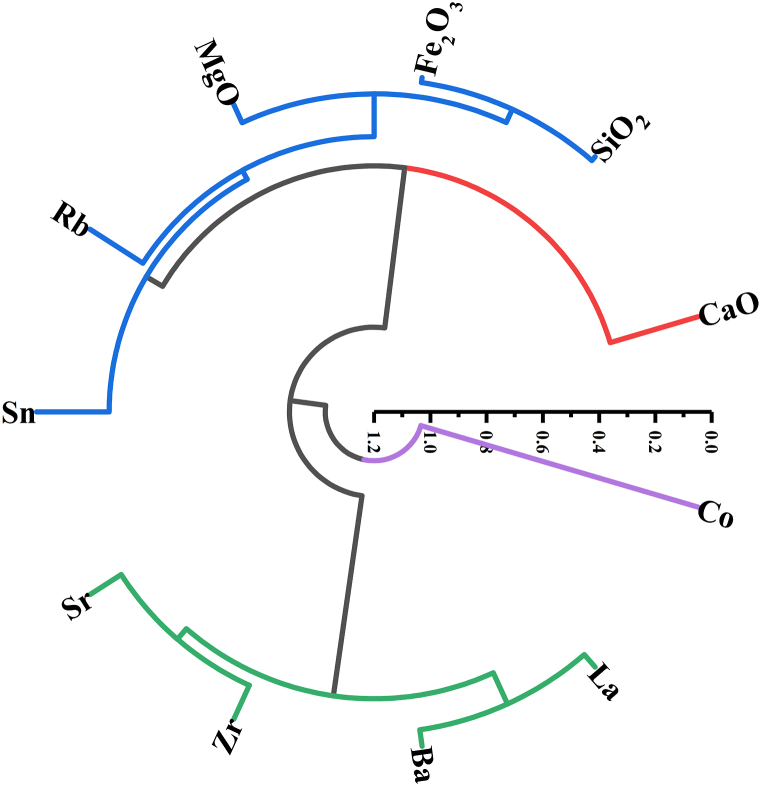
Cluster analysis dendrogram in R-mode (variables).

Among the trace elements, Rb and Sn show significant positive correlations with CaO, SiO_2_, Fe_2_O_3_, and MgO. In addition, Ba and La show moderate to low correlations with major oxides (Table 1). By using cluster analysis, the interrelationships between the elemental compositions of the studied gypsum are clarified (Fig. 6). SiO_2_, Fe_2_O_3_, MgO, Rb, and Sn are closely related, representing external flux contributions. The absence of association between Sr, Zr, Ba, and La representing brine elements and CaO may be attributed to the substitution behavior of these elements for Ca in the gypsum lattice. Moreover, the weak and moderate positive correlations among Ba–CaO, Ca–SiO_2_, Ba–Fe_2_O_3_, Ba–MgO, La–SiO_2_, La–Fe_2_O_3_, and La–MgO (Table 1) indicate that terrestrial and marine sources most likely contributed to the Ba and La origin.

### 4.3 Strontium, sulfur, and oxygen isotope systematics

**Fig. 7:**
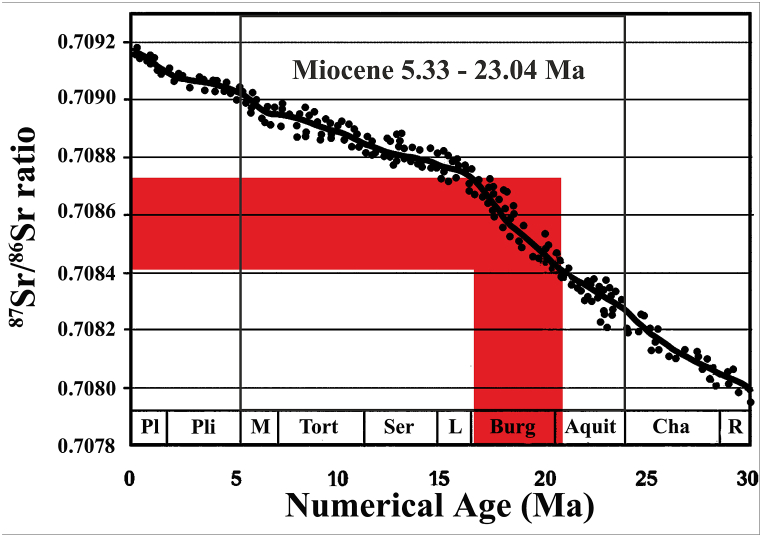
Plots of Miocene Abu Dhabi gypsum ^87^Sr/^86^Sr ratios (red) on variation curve of seawater Sr isotopic composition from Early Oligocene-Holocene interval [52]. (For interpretation of the references to colour in this figure legend, the reader is referred to the Web version of this article.)

The results of ^87^Sr/^86^Sr, δ^18^O_VSMOW_, and δ^34^S_VCDT_ isotope analyses are shown in Table 2. The Sr isotopic ratios (^87^Sr/^86^Sr) vary from 0.708411 to 0.708739 (mean = 0.708617) for the analyzed gypsum samples. These values are close to those of the Miocene marine sulfates (∼0.7082–0.7090; [51]), indicating the marine origin of this facies. Regarding the anticipated Lower Miocene stratigraphic age of these evaporite facies the ^87^Sr/^86^Sr ratio fit to the marine Sr isotope curve of McArthur et al. [52] for the time interval of ∼21.12–15.91 Ma numeric ages (Late Aquitanian-Burdigalian) (Fig. 7).

**Table 2:** Isotopic composition of Abu Dhabi evaporite rocks (N = 18).

No.	Rb/Sr	^87^Sr/^86^Sr	±2σ	Numerical age (Ma)	δ^18^O_VSMOW_‰	δ^34^S_VCDT_‰
1	0.0085	0.708724	8.79E-06	16.15	19.16	21.59
2	0.0194	0.708595	7.94E-06	18.21	14.89	17.10
3	0.0190	0.708739	8.65E-06	15.91	15.51	18.56
5	0.0175	0.708411	8.08E-06	21.12	14.51	18.40
6	0.0180	0.708499	7.65E-06	19.72	14.62	17.63
7	0.0099	0.708718	8.50E-06	16.26	15.25	17.68
8	0.0009	0.708547	8.08E-06	18.96	13.79	20.42
9	0.0006	0.708509	8.36E-06	19.57	15.28	19.37
12	0.0140	0.708648	7.94E-06	17.37	13.06	20.57
13	0.0138	0.708722	8.65E-06	16.19	13.45	20.05
14	0.0078	0.708644	9.64E-06	17.43	13.02	20.93
15	0.0104	0.708653	7.09E-06	17.29	13.70	20.49
16	–	–	–	–	15.44	17.22
17	–	–	–	–	12.92	21.09
18	–	–	–	–	13.71	17.06
19	–	–	–	–	11.88	20.04
20	–	–	–	–	13.23	21.51
21	–	–	–	–	13.87	17.84
Min	0.0006	0.708411	7.09E-06	15.91	11.89	17.10
Max	0.0194	0.708739	9.64E-06	21.12	19.16	21.59
Mean	0.0116	0.708617	8.28E-06	17.87	14.29	19.31

**Fig. 8:**
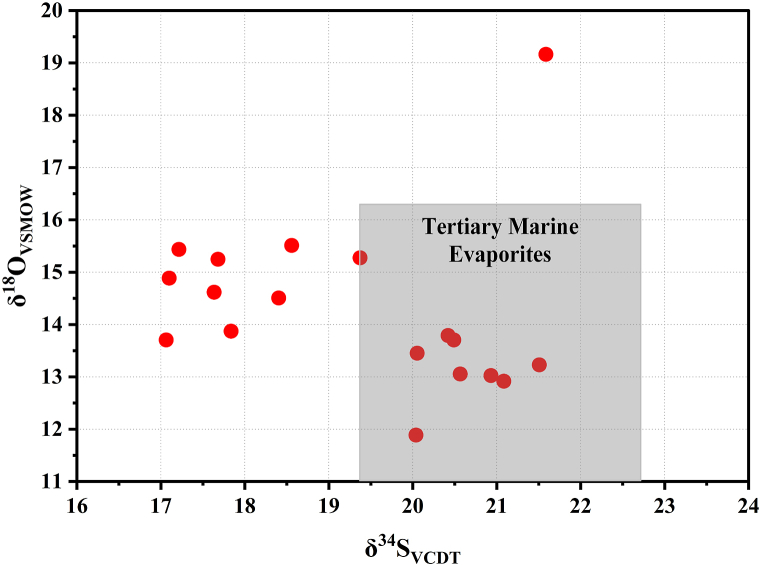
δ^34^S versus δ^18^O cross-plot for the studied gypsum (isotopic values for Tertiary marine evaporites comes from Utrilla et al. [54] and Moragas et al. [20]).

The δ^34^S values fall in the range of 17.10‰). −21.59‰ (mean 19.31‰). These values are relatively lower than the supposed value of Miocene seawater (21‰–23‰; [53]) and Tertiary seawater (19.4‰–22.7‰; [54]) (Fig. 8). Due to the limited significance of isotope fractionation associated with the precipitation of sulfates, the δ^34^S values of evaporitic minerals should reflect the isotope composition of fluids from which they precipitate [53,55,56]. The sulfate isotope composition does not change during gypsum deposition and anhydrite hydration, owing to the negligible sulfur isotope fractionation [4,18,45,57]. In addition, the effect of bacterial sulfate reduction should increase the δ^34^S recorded values [21,23]. The recorded relatively low δ^34^S values may be due to the mixing of the marine brines with a very subordinate flux of non-marine fluids enriched with ^32^S.

The δ^18^O values range between 11.89‰ and 19.16‰ (mean 14.29‰). These values are comparable to those in Upper Burdigalian Vilob Gypsum from Spain [20]. The observed changes in δ^18^O values may have been influenced by the geomorphological position of the basin of deposition (coastal sabkha) [7], as well as the geographical location; notably, the highest recorded δ^18^O values were found in sample No. 1, which was the closest sample to the modern sabkha (Fig. 1b). The positive δ^18^O values indicate that these evaporite facies precipitated in high evaporation rate sabkha under dry climatic conditions [7]. The δ^34^S and δ^18^O isotopic compositions of Abu Dhabi gypsum suggest the marine origin of the brines (Fig. 8).

On the basis of the presented data set, depositional facies, early and late diagenetic evidence, and variation in δ^34^S and δ^18^O, two questions remain open: What is the exact age of the deposition of this evaporite facies (Gachsaran Formation)? And To what extent do diagenetic processes affect the age?

Various screening results have shown that the secondary gypsum underwent diagenetic alterations (hydration and dehydration). The impact of diagenetic alteration is to shift the marine stable isotope signature to a higher or lower value, depending on the source of fluids during diagenesis [12]. Fundamentally, the ability to obtain reliable data from only unaltered minerals necessitates a deep understanding of diagnostic processes. In addition, detrital material may also function as a contamination source, changing the Sr isotope ratio and resulting in biased results. Radiogenic ^87^Sr in detrital grains may exchange with marine Sr during diagenesis [12,19]. However, the investigated evaporite samples contain only a trace of detrital material (clay minerals and quartz). Because of the low Rb/Sr ratios, radiogenic ^87^Sr appeared to have no impact on the gypsum samples analyzed. Thus, the Sr isotope composition of gypsum can represent the original brine Sr isotopic signature where gypsum precipitates [23]. Moreover, the measured ^87^Sr/^86^Sr ratios are identical for the Miocene marine seawater (∼0.7082–0.7090; [51]). The marine Sr signal is mostly preserved in the various post-depositional infillings, in contrast with the δ^18^O and, to a lesser extent, δ^34^S isotope signals, which change depending on the origin and temperature of the water flowing through the fracture sets [20,21,58]. The absence of a constant and adequate supplement of seawater to the closed evaporites basin may typically cause a ±2 fluctuation in δ^34^S values [21]. During diagenetic alterations, the fractionation of S isotopes between sulfate minerals and dissolved sulfate is small [4,59]. Freshwater flux in coastal and shallow basins did not have a measurable influence on ^87^Sr/^86^Sr ratios until salinity was extremely low (10 ppt or less) [13]. Gypsum precipitated from hybrid brines has a wide range of ^87^Sr/^86^Sr ratios [12,60]. The impermeable nature of gypsum and anhydrite inhibits external fluid interactions, and the resorption of post-depositional waters via anhydrite rehydration could hardly cause a significant variation in the ^87^Sr/^86^Sr ratio [12]. The estimated age based on the ^87^Sr/^86^Sr ratio (Late Aquitanian-Burdigalian) appears to be correct and unaffected by diagenetic processes or contaminations by the scarce continental flux that may affect sulfur and oxygen isotopes.

Another relevant point that supports the Gachsaran Formation estimated age is the measured age of the underlaying and overlaying formations. The deposition of the Asmari Formation in the UAE onshore extended to the beginning of the Aquitanian, whereas offshore, it extended to the Burdigalian [36]. Paleomagnetic pole position dating of the Shuweihat Formation has an approximate age of 15 Ma [34].

## 5 Conclusions

A multidisciplinary approach enabled us to improve the knowledge of the subsurface Miocene evaporitic facies in Abu Dhabi. The petrographic and mineralogical examination indicates that these evaporite facies were formed in a sedimentary environment with weak hydrodynamic conditions and low input from terrigenous material. The effect of early diagenesis (hydration and dehydration) on the formation of secondary gypsum was revealed by the different gypsum textures. The geochemical composition indicates similar physicochemical conditions with relatively low salinity. Extrabasinal detrital contribution has a noteworthy influence on the distribution of the recorded low concentrations of trace elements. The elevated Sr concentrations in some samples have been linked to the presence of celestite minerals. The sulfur and oxygen isotope compositions confirm that they underwent diagenetic processes and were influenced by geomorphological and geographical position, as well as by the flux of non-marine fluids. Nevertheless, the ^87^Sr/^86^Sr ratio provides convincing age results, indicating that the Sr isotopic ratio is unaffected by diagenetic processes or the scarce continental flux that affects sulfur and oxygen isotopes. The subsurface Miocene evaporite facies (Gachsaran Formation) in Abu Dhabi formed in coastal salina/sabkha under dry climatic conditions in a time interval of 21.12–15.91 Ma (Late Aquitanian-Burdigalian). The geochemical and isotopic data of the Abu Dhabi Miocene evaporites presented here may represent a significant contribution for a better understanding of the origin of ancient evaporites in the Arabian Gulf Basins.

## Author contributions statement

**Ahmed Gad:** conceived and designed the experiments; analyzed and interpreted the data; contributed reagents, materials, analysis tools or data; wrote the paper. **Osman Abdelghany:** conceived and designed the experiments; performed the experiments; analyzed and interpreted the data; contributed reagents, materials, analysis tools or data; wrote the paper. **Hasan Arman:** conceived and designed the experiments; performed the experiments; analyzed and interpreted the data; contributed reagents, materials, analysis tools or data; wrote the paper. **Bahaa Mahmoud:** conceived and designed the experiments; performed the experiments; analyzed and interpreted the data; contributed reagents, materials, analysis tools or data. **Ala Aldahan:** conceived and designed the experiments; analyzed and interpreted the data; contributed reagents, materials, analysis tools or data; wrote the paper. **Safwan Paramban:** conceived and designed the experiments; performed the experiments; contributed reagents, materials, analysis tools or data. **Mahmoud Abu Saima:** conceived and designed the experiments; performed the experiments; analyzed and interpreted the data; contributed reagents, materials, analysis tools or data; wrote the paper.

## Funding statement

Dr. Hasan Arman was supported by The 10.13039/501100006013United Arab Emirates University, Research Affairs National Water and Energy Center, NWC–4–2018–31R193.

## Data availability statement

Data will be made available on request.

## Declaration of competing interest

The authors declare that they have no known competing financial interests or personal relationships that could have appeared to influence the work reported in this paper.
